# The circRNA Landscape in Recurrent Pregnacy Loss (RPL): A Comparison of Four Reproductive Tissues

**DOI:** 10.3390/ijms252312622

**Published:** 2024-11-25

**Authors:** Endika Varela-Martínez, Olaia Colau, Renate G. van der Molen, Begoña M. Jugo

**Affiliations:** 1 Department of Genetics, Physical Anthropology and Animal Physiology, Faculty of Science and Technology, University of the Basque Country (UPV/EHU), Sarriena auzoa, 48940 Leioa, Spain; endika.varela@ehu.eus (E.V.-M.);; 2 Department of Laboratory Medicine, Laboratory of Medical Immunology, Radboud University Medical Center, 6500 HB Nigmegen, The Netherlands

**Keywords:** RPL, RNAseq, circRNA, decidua, chorionic villus, endometrium

## Abstract

Recurrent Pregnancy Loss (RPL), also named Recurrent Spontaneous Abortion (RSA), is a common fertility problem that refers to at least two consecutive pregnancy losses and affects 1–2% of couples all over the world. Despite common causes such as genetic abnormalities, uterine anomalies or hormonal and metabolic disorders, there is still a huge challenge in identifying the causes of about 40–60% of RPL patients. Circular RNAs (circRNAs) are endogenous ncRNAs with a unique closed-loop and single-stranded structure. Accumulated evidence indicates the role of circRNAs in embryonic development and implantation, which may help decipher the mechanisms and causes underlying RSA. Four works were selected in the SRA public repository that used RNAseq analysis in control and RPL samples in four tissues: endometrium, chorionic villus tissue, decidua and decidua immune cells. Two programs were selected for circRNA detection: DCC and CIRI2. A total of 1715 candidate circRNAs were detected after filtering the results. In the differential expression analysis, decidual tissue showed the highest percentage of circRNA with differential expression between cases and controls. CircRNAs originating from genes *OGA*, *FNDC3B*, *RAB11FIP1*, *SIPA1L2* and *GREB1L* showed the highest expression in women suffering from pregnancy losses, in decidual tissue or endometrium. In the GO term enrichment analysis, multiple terms related to embryonic development and immunological response were consistently enriched in villus and decidual tissues. Although some differentially expressed circRNAs were shared between tissues, decidua seems the tissue of choice for analyzing the role of circRNAs in RPL.

## 1. Introduction

Recurrent Pregnancy Loss (RPL), also named Recurrent Spontaneous Abortion (RSA), is a common fertility problem that affects 1–2% of couples all over the world [1]. RPL refers to at least two consecutive pregnancy losses before reaching viability according to the European Society for Human Reproduction and Embriology [2]. The etiology of RPL may be multifactorial and diverse among patients. The most common causes include genetic abnormalities, uterine anomalies, antiphospholipid syndrome, hormonal and metabolic disorders, and increasing maternal age. Despite the array of causes listed above, there is still a huge challenge in identifying the causes of about 40–60% of RPL patients [3], which are often referred to as unexplained recurrent pregnancy loss patients.

Circular RNAs (circRNAs) are a new class of endogenous ncRNAs with a unique closed-loop and single-stranded structure which results in a longer half-life. They can act as miRNA sponges to regulate the function of miRNAs, as transcription or translation regulators to affect protein expression and can also interact with proteins to regulate gene expression. Accumulated evidence indicates the role of circRNAs in embryonic development and implantation, which may help decipher the mechanisms and causes underlying RSA. Li et al. (2020) [4], for example, found that compared with women with normal pregnancies, 123 differentially expressed circRNAs were found in patients with early RSA, including 78 upregulated and 45 downregulated circRNAs. On the other hand, Liu et al. (2022) [5] detected 22 circRNAs differentially expressed in an RSA group.

The exponential increase in RNA sequencing datasets in recent years offers a valuable opportunity for posing novel scientific questions or improving the statistical significance of analyses in a cost-efficient manner [6].

The present project’s main aim is to detect and characterize the main non-coding RNAs involved in the Recurrent Pregnancy Loss (RPL) female reproductive disorder in different tissues. To this end, RNA-seq studies from repositories were recovered. Studies from four different tissues or cells could be found: endometrium, chorionic villus tissue, decidua and decidua immune cells. All of them were analyzed to detect circRNAs following the same bioinformatic procedures in order to make the results comparable among tissues.

## 2. Results

### 2.1. RNA-Seq Data Quality

The average sequencing depths in million reads in each project were the following: 31.57 (±1.16 SD) in endometrium; 41.74 (±1.81 SD) in decidua immune cells; 48.30 (±1.96 SD) in decidua; and 53.12 (±7.45 SD) in villus. After adapter removal and quality filtering with Trimmomatic, between 94.19% (decidua) and 97.63% (decidua immune cells) of the sequences remained. From those sequences retained after filtering, the percentage of reads that could be assigned to rRNA genes by bbduk ranged from 0.75% (villus) to 7.56% (endometrium). It was confirmed later, after quantification of gene expression, that few sequences could be assigned to rRNA genes. The reads that passed the previous filtering steps were aligned to the reference genome. The mapping performed by STAR had the following average alignments: from 93.41% (endometrium) to 95.22% (decidua) of the reads that remained after filtering could be assigned uniquely to the reference; from 4.05% (decidua) to 5.93% (endometrium) of the reads could be aligned to multiple loci; and 0.26% (endometrium and decidua) to 0.59% (decidua immune cells) were classified as chimeric reads with at least two segments mapping to different loci of the reference in a non-linear manner. For a more detailed description of the quality filtering and alignment for each sample, see Supplementary Table S1.

### 2.2. CircRNA Characterization

The data were analyzed by DCC and CIRI2 for circRNA back-splice junction (BSJ) identification. Prior to any filtering, 92,999 and 19,030 candidate circRNAs were identified by DCC and CIRI2, respectively. Out of those candidate circRNAs, 16,374 were detected by both tools. A circRNA was taken as expressed in the tissue if it had at least five supporting reads in half of the samples. A total of 1715 circRNAs remained for subsequent analyses. In each tissue, the proportion of circRNAs detected was different (Table 1), with the chorionic villus tissue having more circRNAs (1113 circRNAs), followed by the decidual tissue (983 circRNAs) and the decidual immune cells (646 circRNAs). The endometrium was the tissue with the lowest number of circRNAs; specifically, only 285 circRNAs were detected. Comparing the detected circRNAs with those collected in the CIRCpedia database, 1581 of the circRNAs had already been detected in previous works. Therefore, 134 new circRNAs were detected in these samples.

**Figure 1:**
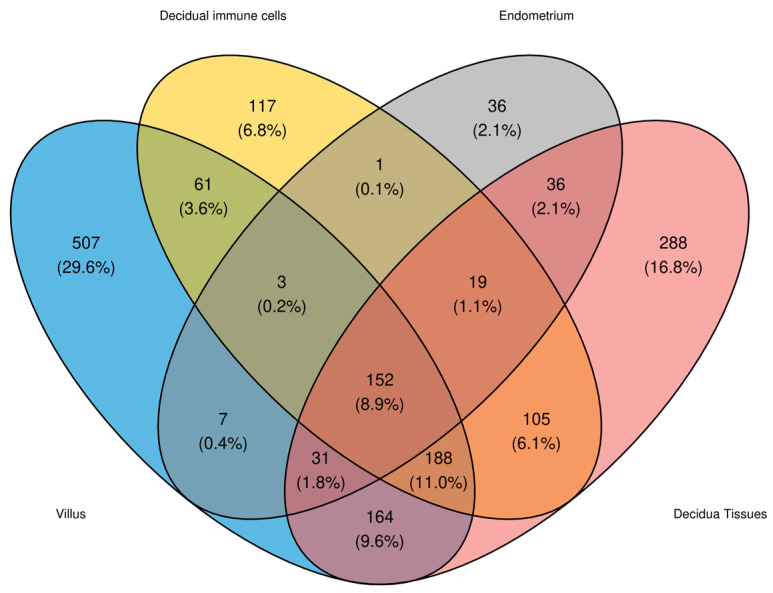
Venn diagram of circRNAs detected in each tissue differentiated by color (blue for villus tissue, yellow for decidual immune cells, gray for endometrium and red for decidual tissue).

Using a Venn diagram, it was observed how many circRNAs were detected in each tissue (Figure 1). In total, 152 circRNAs were in common in all four tissues. A total of 29.6% of the circRNAs detected were found exclusively in villus tissue, and 16.8% and 6.8% in decidual tissues and 2.1% in endometrium, so the villus tissue presented the highest number of specific circRNAs.

The detected circRNAs were spread across all chromosomes (Supplementary Figure S1A), and it was observed that the longer the chromosome, the more circRNAs were detected. At the gene level, we saw that Most of the circRNA-producing genes produce only one circRNA (Supplementary Figure S1B).

Looking at the evolutionary conservation of these BSJs compared with those described in mice, 772 (45.01%) homologous and 361 (21.05%) non-homologous circRNAs were detected (Supplementary Figure S2). 

### 2.3. Differential Expression Analysis

**Figure 2:**
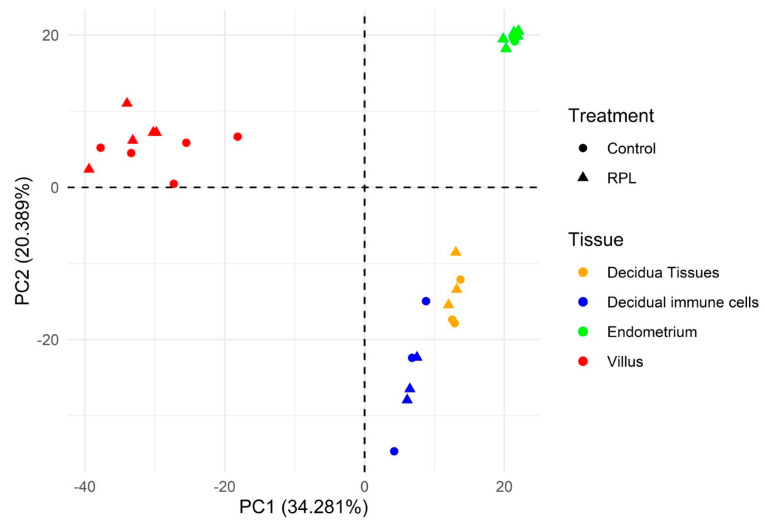
PCA result. Each color represents a different tissue (red for villus tissue, blue for decidual immune cells, green for endometrium and blue for decidual tissue); the dots are the controls and the triangles are the RPL samples.

In the Principal Component Analysis (PCA), the samples of the different projects (Figure 2) were distributed in three groups, corresponding to the different types of tissue that have been analyzed: villus tissue, endometrial tissue and decidual tissue (along with decidual immune cells). The endometrium and the decidua correspond to the human uterine mucosa in non-pregnant and pregnant states, respectively.

In the differential expression analysis of the 1113 circRNAs detected (in Table 2, the top 20 differentially expressed circRNAs are shown; all of them are in Supplementary Table S2), only 4 (0.36%) showed differential expression between cases and controls in the villus tissue, all of them being underexpressed; 8 circRNAs (2.8% of the circRNAs) in the endometrial tissue, 5 overexpressed and 3 underexpressed; 41 circRNAs (6.35% of the circRNAs) in immune cells from the decidual tissue, 14 overexpressed and 27 underexpressed; and 123 (12.5% of the circRNAs) in the decidual tissue, 14 overexpressed and 109 underexpressed. CircRNAs originating from the genes *KCNN2*, *FAT3*, *ACAP2*, *SNORD113* and *BTBD10* presented the highest differential expression, with the expression being lower in women with RPL. Finally, very few differentially expressed circRNAs were shared between tissues.

### 2.4. Functional Analysis

**Figure 3:**
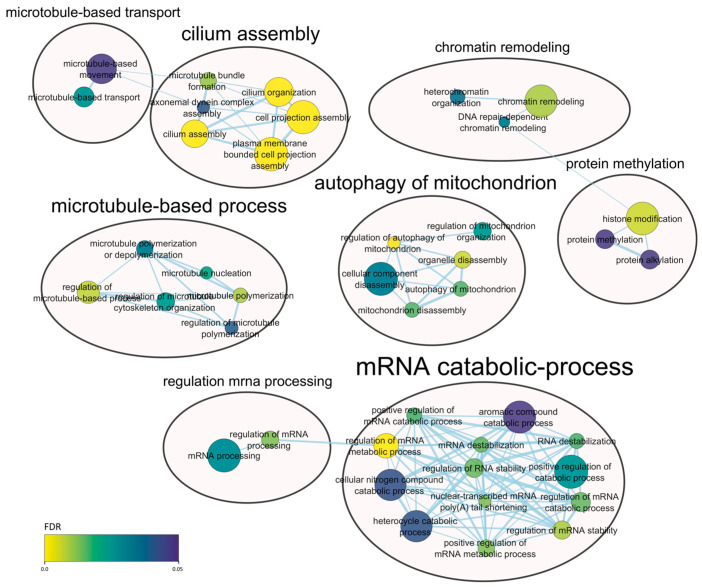
Biological processes described in circRNA-based functional analysis detected in endometrium tissue. If the same gene appears in different functions, these are connected with blue lines. The size of the node depends on the number of genes summed by each term (the more genes, the bigger the size) and the color of the node indicates the degree of FDR significance (from 0 to 0.05).

Taking into account the genes of origin of the detected circRNAs in each tissue, functional analyses were performed. Among the enriched terms in endometrium (Figure 3), there were general terms related to mRNA processing such as regulation of mRNA stability (FDR = 8.77 × 10^−3^), regulation of mRNA catabolic process (FDR = 1.47 × 10^−2^), mRNA destabilization (FDR = 1.47 × 10^−2^) and nuclear-transcribed mRNA poly(A) tail shortening (FDR = 1.28 × 10^−2^), among others.

**Figure 4:**
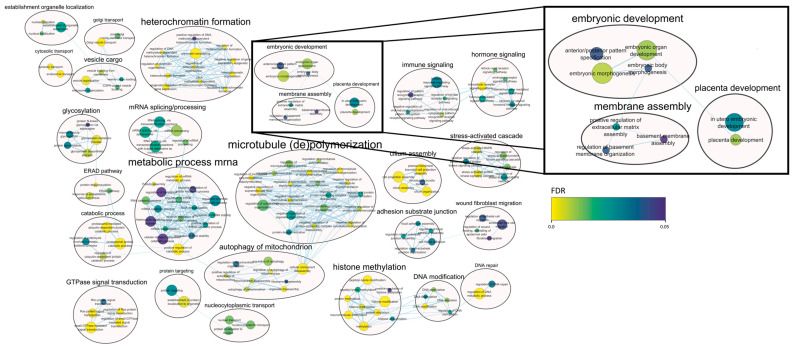
Biological processes described in circRNA-based functional analysis detected in decidual tissue. If the same gene appears in different functions, these are connected with blue lines. The size of the node depends on the number of genes summed by each term (the more genes, the bigger the size) and the color of the node indicates the degree of FDR significance (from 0 to 0.05).

Regarding decidua tissue (Figure 4), there were terms related to placental and embryonic development such as embryonic morphogenesis (FDR = 7.63 × 10^−3^), embryonic organ development (FDR = 1.03 × 10^−2^) and placenta development (FDR = 1.02 × 10^−2^); terms related to the immunological response such as regulation of the toll-like receptor 9 signaling pathway (FDR = 1.86 × 10^−2^) and regulation of the pattern recognition receptor signaling pathway (FDR = 4.61 × 10^−2^); stress-related terms such as the stress-activated MAPK cascade (FDR = 5.03 × 10^−3^), JNK cascade (FDR = 1.30 × 10^−2^) and stress-activated protein kinase signaling cascade (FDR = 2.66 × 10^−3^); and terms related to catabolic processes such as regulation of proteolysis involved in protein catabolic process (FDR = 3.00 × 10^−2^), mRNA catabolic process (FDR = 2.93 × 10^−2^) and heterocycle catabolic process (FDR = 4.74 × 10^−2^), among others.

In the decidual immune cells (Supplementary Figure S3), there were terms related to placental and embryonic development, detected also in decidua tissue, such as placenta development (FDR = 7.48 × 10^−3^), somitogenesis (FDR = 2.44 × 10^−2^), embryonic organ development (FDR = 2.59 × 10^−2^) and cranial skeletal system development (FDR = 4.40 × 10^−2^); terms related to the immunological response such the T cell receptor signaling pathway (FDR = 2.50 × 10^−2^) and antigen receptor-mediated signaling pathway (FDR = 2.05 × 10^−2^); terms related to catabolic processes such as positive regulation of the mRNA catabolic process (FDR = 1.35 × 10^−4^) and regulation of the proteasomal ubiquitin-dependent protein catabolic process (FDR = 2.50 × 10^−2^); and stress-activated signaling terms such as regulation of the stress-activated MAPK cascade (3.66 × 10^−2^) and regulation of the p38MAPK cascade (2.86 × 10^−3^). Finally, villus shared multiple terms seen in decidual immune cells and decidua (Supplementary Figure S4); there were terms related to placental development and embryonic morphogenesis such as placenta development (FDR = 5.98 × 10^−3^), but it presented other specific terms such as spongiotrophoblast layer development (FDR = 4.76 × 10^−2^), embryonic organ morphogenesis (FDR = 2.85 × 10^−2^), embryonic skeletal system development (FDR = 1.95 × 10^−2^), cranial skeletal system development (FDR = 1.78 × 10^−2^) and neural tube development (FDR = 5.56 × 10^−3^). Moreover, some other similar terms for the decidual immune cells related to immunological response arose, such as the T cell receptor signaling pathway (FDR = 3.16 × 10^−2^) and antigen receptor-mediated signaling pathway (FDR = 4.63 × 10^−2^); shared terms related to catabolic processes such as positive regulation of the mRNA catabolic process (FDR = 2.89 × 10^−4^) and regulation of the proteasomal ubiquitin-dependent protein catabolic process (FDR = 7.90 × 10^−4^) arose; and other terms such as cellular response to transforming growth factor beta stimulus (FDR = 1.50 × 10^−2^) and multiple terms related to hindbrain morphogenesis and telencephalon development, among others, arose. 

## 3. Discussion

In the present study, re-analysis of rRNA-depleted RNA-seq libraries of RPL and control samples from public repositories was performed to characterize circRNAs. The main problems arising when working with these ncRNAs are the limited information when reporting results, the lack of a standard nomenclature for naming circRNAs and the differences in pipelines, so comparisons between studies are usually hard to perform. In order to overcome these limitations, in this work, all samples were analyzed with the same pipeline. This procedure avoids differences in quantification due to the use of different tools and filtering criteria, which can affect downstream differential expression analyses [7]. Regarding sample size, the total number of samples analyzed in this work was 31: 14 controls and 17 from women with RPL. However, they were obtained from four different tissues and were analyzed separately, so the analysis of more samples would be desirable.

In this work, 1715 candidate circRNAs were characterized in RPL and control samples, of which endometrium expressed the lowest number and chorionic villus the highest: 285 and 1113, respectively. However, due to the different sequencing depth in the samples from different projects, we cannot completely discern if this difference is due to the sequencing depth itself or to different expression of circRNAs in different tissues. Only circRNA junction spanning reads are used in the analysis for circRNA expression trends in total RNA-seq datasets and those reads comprise less than 0.1% of the reads in a typical total RNA-seq experiment [8]. Thus, a snapshot of highly expressed circRNAs is seen in endometrium samples and those samples would gain from a higher sequencing depth. In general, in two of the previous studies, a higher number of circRNAs were detected compared with our results. For example, in the original work of Liu et al. [9], they detected 5126 circRNAs whilst we detected 983. This can be due to the application of stricter expression level filters in the bioinformatics analysis of this work. The characteristics of the detected circRNAs, such as chromosomal distribution and frequency, the number of circRNAs per gene and the number of exons per circRNA, are typical [10]. From the 1715 circRNAs detected in this work, 1581 were already annotated in the CIRCpedia database. Thus, 134 new circRNA candidates were detected.

We have observed that tissue of origin is the main factor determining circRNA expression patterns. Three clusters of samples were defined in the PCA: endometrium, chorionic villus and decidua (cluster composed of decidua tissue and decidual immune cell samples). Chorionic villi make up a significant portion of the placenta and serve primarily to increase the surface area by which products from the maternal blood are made available to the fetus. Chorionic villus cells contain the same genetic material as that of the fetus. That is the reason why cells of the chorionic villi can be collected and examined to determine whether a fetus is affected by a genetic disorder. Its origin can explain the differentiation from the other samples in the PCA, and the low number of significant differentially expressed circRNAs. As for the decidual and endometrial tissues, they belong to the same anatomical part of the uterus, although in different vital phases. In recent single-cell studies of endometrium and endometrium during decidualization in human [11] and mouse [12], it was observed (1) that the cell population is quite heterogeneous, and (2) the accumulation of more immune cells in decidua compared to endometrium.

In relation to the number of differentially expressed circRNAs in villus, only 4 differentially expressed circRNAs were detected in this study, whilst 163 were found in the original paper. Again, this can be due to the application of stricter expression level filters in this work. Moreover, a number of differentially expressed circRNAs that have been described in previous studies were also detected in this study, such as circRNAs originating from *SCAF11*, *UBAP2*, *EPS15* and *PTPN12* genes [13]; *EPSTI1* and *PICALM* [14]; and *MYOF* [5], all of them expressed in decidua or decidua immune cells. The circRNA *hsa-circUBAP2-002*, which we found with decreased expression in decidual tissue, merits special attention, as decreased expression of a circRNA originating from the *UBAP2* gene has been associated with preeclampsia by limiting trophoblast cell proliferation and migration by Qi et al. (2021) [15].

CircRNAs originating from the genes *KCNN2*, *FAT3*, *ACAP2*, *SNORD113* and *BTBD10* present the biggest difference in expression, with the expression being lower in women with RPL. All of them, except *BTBD10*, were present in the villus tissue. On the other hand, circRNAs originating from *OGA*, *FNDC3B*, *RAB11FIP1*, *SIPA1L2* and *GREB1L* showed the highest expression in women suffering from pregnancy losses, expressed in decidual tissue or endometrium. Among these five genes, only the *OGA* gene has been previously linked to miscarriages and embryo developmental failure; according to de Lima Castro et al. (2023) [16], several processes such as trophoblast differentiation and placental vasculogenesis are related to this gene. The four genes newly associated with RPL and their circRNAs require more in-depth analyses.

When comparing the differentially expressed circRNAs among tissues, only *hsa_circRNA_SPECC1_001* and *hsa_circRNA_EMILIN_001* were shared by endometrial and decidual tissues, and both show homologous conservation in mice. The *SPECC1* gene has recently been linked to blastocyst development in mouse [17] and the *EMILIN* gene has been associated with elastic fibers. Braghetta et al. [18] have suggested that *EMILIN* may be involved in placental development and subsequent organogenesis in mouse. Both circRNAs could perform similar tasks in humans also. The remaining differentially expressed circRNAs detected in endometrium and decidual tissue and its immune cells are completely different, which could reflect the process of decidualization.

In relation to the GO term enrichment analyses with all the circRNAs expressed in each tissue, multiple terms related to embryonic development and immunological response were consistently enriched in villus and decidual tissues. On the other hand, in a recent article that analyzes circRNA expression in decidua samples with RPL [19], there were terms related to regulation of GTPase activity, chromatin modification, histone modification, vesicle localization and vesicle targeting among the most enriched terms for the differentially expressed circRNAs. In both the decidua and villus tissues analyzed in this work, such terms were also found among the most significant ones. Finally, the mitogen-activated protein kinase (MAPK) has been related to the regulation of trophoblast invasion and migration in villi from patients of RPL [20] and in this work, terms related to the MAPK cascade were found in the tissues of decidual origin. It seems that decidual tissues and villi share significantly enriched terms. The circRNAs related to those terms may require more in-depth analyses.

Other authors [21] propose that circRNAs may eventually be used as biomarkers for RPL. Among the four compared tissues, decidua shows the more differentially expressed circRNAs, *hsa-circEMILIN2-001* being one of them. Although some differentially expressed circRNAs are shared between decidua and other tissues, decidua seems the tissue of choice to analyze the role of these non-coding RNA in RPL. We are now beginning to disentangle the relationship between circRNAs and reproductive diseases.

## 4. Materials and Methods

### 4.1. Database Selection and Quality Control

**Figure 5:**
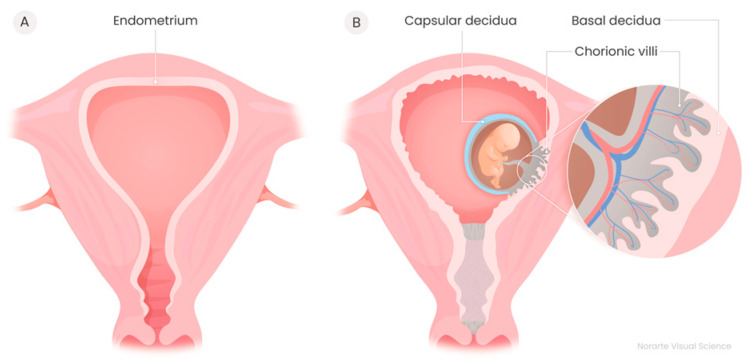
(**A**) Human uterus. (**B**) Human uterus at pregnancy.

The Sequence Read archive (SRA) public repository was used to search for samples for our analyses. The data for the analyses were searched using the following keywords: “RNAseq” and four different terms to name the disease, namely Unexplained Recurrent Spontaneous Abortion (URSA), Recurrent Pregnancy Loss (RPL), Recurrent Spontaneous Abortion (RSA) and Recurrent Miscarriage (RM). The term RPL was chosen to be used through this article for consistency. From this search, we found 4 articles that used RNAseq libraries with an rRNA reduction step for Total RNA sequencing. In two of the articles, PRJNA760763 project in chorionic villi tissue [22] and PRJNA819201 project in decidua tissue [9], circRNAs had already been detected. The other two articles, which only used the data of protein-coding genes for differential expression analyses, were performed on the immune cells of decidua tissue in the PRJNA813430 project [23] and on the endometrium tissue on the 7th day after luteal hormone surge in the PRJNA314429 project [24]. The tissues analyzed are represented in Figure 5. Samples of the four studies were taken from women younger than 40 years old and detailed clinical information is covered in each original article. 

In this work, all RNAseq samples were re-analyzed following the same pipeline to avoid batch effects due to the use of different tools and filtering criteria. Prior to the characterization of circRNAs, the quality of the samples was checked. A schematic representation of the full circRNA characterization pipeline is shown in Figure 1. First, the data from the selected four projects were downloaded with SRA toolkit. FastQC [v0.11] and MultiQC [v1.15] were used to check the quality of the raw data. Then, the trimming of low-quality sequences and adapters was performed with Trimmomatic [v0.39] using the following criteria: (1) trimming of bases from the start or end of a read if their quality phred value was below 20; (2) trimming if the average quality within a sliding window of five nucleotides was below 20; and (3) read filtering if their length after trimming was sorter than 40. After that, residual rRNA was removed using BBDUK [v39.1] and SILVA database, and another quality control was applied. 

### 4.2. CircRNA Identification

For circRNA identification, two tools were selected, detect circRNAs from chimeric reads (DCC) [v0.5.0] and CircRNA Identifier 2 (CIRI2) [v2.0]. Before running DCC, quality-filtered reads were aligned to the human reference genome (GRCh38), downloaded from Ensemble [release-109], with Spliced Transcripts Alignment to a Reference (STAR) [v2.7.1a] following DCC author recommendations. Then, RSeqQc [v5.0.1] and Qualimap [v2.2.1] were used to check the quality of the alignments. DCC was run with the annotation file from the reference genome and a file with repetitive regions from the University of California Santa Cruz (UCSC) genome browser (Repeat Masker and Simple Repeats tracks) and with default parameters, except that we required that a circRNA had to be expressed with one read in at least one sample to be reported. In contrast, for CIRI2, quality-filtered reads were aligned to the human reference genome using BWA-MEM [v0.7.17] and CIRI2 was run with default parameters. Then, back-splice junction (BSJ) read detection was performed by re-alignment of reads against a pseudo-circular reference constructed from the BSJ detected by CIRI2 with the CIRIquant module. After that, an intersection of the BSJs identified in the two software was performed using R [v4.1.1] and the quantification performed by CIRIquant was used for further analyses. 

To name the circRNAs detected, the nomenclature rules described in [25] were followed with some slight modifications. Specifically, to name the detected exonic and intronic circRNAs, the codes hsa-circHUGO-# and hsa-ciHUGO were used, respectively, where HUGO refers to the name of the gene of origin of each circRNA and the # is a three-digit number that is assigned sequentially. In the case of intergenic circRNAs, hsa-circChrom#-# was used, the first # being the number of the chromosome in which the circRNA is located. 

### 4.3. Evolutionary Conservation Analysis

The detected circRNAs were compared to the ones annotated in CIRCpedia for mice (*Mus musculus*). The analysis was carried out following the steps from Varela-Martínez et al. [26]. First, the 5′ and 3′ coordinates of each circRNA found were converted to mouse coordinates with the USCS liftOver v1.30.0 tool. The resulting coordinates were screened for overlap with mouse annotated circRNAs in CIRCpedia. Splice sites detected in ±2 nt intervals around the putative mouse sites were considered homologous. And different categories were assigned to each circRNA: “not-aligned”, the human coordinates were not translated to mouse with liftOver; “no homologous”, no mouse circRNA detected near both splice sites; “5′ site utilized”, a mouse circRNA that only uses the 5′ splice site is detected; “3′ site utilized”, a mouse circRNA that only uses the 3′ splice site is detected; “both sites utilized”, both splice sites are used by different circRNAs in mouse; and “homologous”, a mouse circRNA using both splice sites is detected. 

### 4.4. Differential Expression Analysis

The quantification from CIRIquant was filtered and normalized with the following criteria: circRNAs with ≥5 counts in half of the samples in each project were taken as expressed, and for normalization with DESeq2 [v3.17], the full size of the library was considered. Prior to the differential expression analysis, a Principal Component Analysis (PCA) was performed to observe the variation between projects and samples (controls and RPL). To analyze the differential expression of the detected BSJs between control and RPL samples, the DESeq2 [v3.17] package of R was used, setting an adjusted *p*-value < 0.05 (adjusted with the Benjamini and Hochberg method) and log2FoldChange > |0.58| as the significance cut-off. 

### 4.5. Functional Analysis

Under the assumption that the detected circRNAs would be related to the biological processes in which their genes of origin participate, functional analysis was made based on the detected circRNAs in each tissue. This analysis was performed using the GO databases in g:profiler2. The set of all expressed genes detected in the total RNA-seq libraries was set as background and those terms with an adjusted *p*-value < 0.05 (adjusted with the Benjamini and Hochberg method) were selected as significant. 

For visualization purposes, the list of enriched GO terms was further analyzed with Cytoscape v3.9.1 using EnrichmentMap v3.3.5 and AutoAnnotate v1.4. Terms composed of more than 400 genes or less than 5 genes were removed from the analysis. EnrichmentMap generates a network in which pathways are visualized as nodes connected with each other if they share genes. Pathways with common genes often represent similar biological processes and are grouped together as sub-networks. Clusters with less than three interconnected nodes were removed. 

## Figures and Tables

**Table 1:** Description of each project (BioProject ID, tissue of origin and the number of samples in each condition). The number of circRNAs detected in each project and the number of differentially expressed circRNAs refer to the results obtained with our pipeline.

Project ID	Tissue	Number of Samples	Controls	RPL	Detected circRNAs	Differentially Expressed circRNAs
PRJNA760763	Chorionic villus tissue	10	5	5	1113	4
PRJNA314429	Endometrium	9	3	6	285	8
PRJNA813430	Decidua immune cells	6	3	3	646	41
PRJNA819201	Decidua tissue	6	3	3	983	123

**Table 2:** List of differentially expressed circRNAs in each tissue and their baseMean, log2FoldChange, LfcSE, stat, *p*value and *p*adj values.

Tissue	circRNA	baseMean	log2FoldChange	LfcSE	stat	*p*value	*p*adj
Villus tissue	*hsa-circKCNN2-001*	3.3 × 10^−7^	−6.8	1.23	−5.6	2.7 × 10^−8^	2.7 × 10^−5^
	*hsa-circFAT3-001*	6.1 × 10^−7^	−5.3	1.11	−4.8	2.0 × 10^−6^	1.0 × 10^−3^
	*hsa-circSNORD113-9-003*	1.0 × 10^−6^	−4.5	1	−4.5	7.2 × 10^−6^	2.4 × 10^−3^
	*hsa-circACAP2-001*	1.5 × 10^−7^	−5.1	1.21	−4.2	2.2 × 10^−5^	5.5 × 10^−3^
Decidual immune cells	*hsa-circGUSBP1-001*	2.6 × 10^−6^	−2.3	0.26	107.2	4.1 × 10^−25^	2.3 × 10^−22^
	*hsa-circSETD3-001*	4.6 × 10^−6^	1.4	0.17	77.1	1.6 × 10^−18^	4.5 × 10^−16^
	*hsa-circUBE3A-001*	2.8 × 10^−6^	−1.6	0.22	64.6	9.3 × 10^−16^	1.7 × 10^−13^
	*hsa-circTTC39C-001*	2.5 × 10^−6^	1.2	0.22	34.7	3.9 × 10^−9^	3.6 × 10^−7^
	*hsa-circSRCAP-001*	5.0 × 10^−7^	−3.3	0.76	32.7	1.1 × 10^−8^	8.6 × 10^−7^
	*hsa-circMETTL3-001*	1.1 × 10^−6^	1.7	0.36	28.9	7.4 × 10^−8^	5.2 × 10^−6^
	*hsa-circMCTP2-003*	1.0 × 10^−6^	−1.7	0.36	27.1	1.9 × 10^−7^	1.2 × 10^−5^
	*hsa-circSFMBT2-001*	4.8 × 10^−6^	−0.7	0.15	20.1	7.5 × 10^−6^	4.2 × 10^−4^
	*hsa-circRNF13-002*	5.0 × 10^−7^	−2.2	0.57	18.9	1.4 × 10^−5^	7.1 × 10^−4^
	*hsa-circITGAX-001*	6.1 × 10^−7^	1.8	0.49	17.4	3.0 × 10^−5^	1.4 × 10^−3^
	*hsa-circEPS15-001*	4.1 × 10^−7^	−2.3	0.65	16.6	4.6 × 10^−5^	2.0 × 10^−3^
	*hsa-circZNF79-001*	2.8 × 10^−7^	−2.9	0.89	15.8	7.2 × 10^−5^	2.9 × 10^−3^
	*hsa-circSTT3B-001*	1.5 × 10^−6^	−1	0.27	15.2	9.9 × 10^−5^	3.7 × 10^−3^
	*hsa-circWSB1-001*	4.9 × 10^−7^	−1.9	0.55	15	1.1 × 10^−4^	3.7 × 10^−3^
	*hsa-circPTPN12-001*	5.0 × 10^−7^	1.8	0.54	14.1	1.7 × 10^−4^	5.7 × 10^−3^
	*hsa-circCCSER2-002*	7.4 × 10^−7^	−1.4	0.4	13.9	1.9 × 10^−4^	6.0 × 10^−3^
	*hsa-circLPAR1-002*	4.4 × 10^−7^	−1.9	0.58	13.8	2.1 × 10^−4^	6.0 × 10^−3^
	*hsa-circCYFIP2-001*	4.2 × 10^−7^	2	0.61	13.3	2.6 × 10^−4^	7.3 × 10^−3^
	*hsa-circRBM39-003*	5.0 × 10^−7^	1.8	0.54	13.2	2.8 × 10^−4^	7.4 × 10^−3^
	*hsa-circSPECC1-001*	2.2 × 10^−6^	−0.8	0.22	13.1	2.9 × 10^−4^	7.4 × 10^−3^
Endometrium	*hsa-circOGA-002*	2.5 × 10^−7^	4.1	1.22	14.4	1.5 × 10^−4^	1.2 × 10^−2^
	*hsa-circSPECC1-001*	2.3 × 10^−6^	1	0.27	14.3	1.5 × 10^−4^	1.2 × 10^−2^
	*hsa-circLPAR3-001*	1.9 × 10^−6^	1.1	0.31	14	1.8 × 10^−4^	1.2 × 10^−2^
	*hsa-circFNDC3B-007*	4.2 × 10^−7^	2.9	1.05	13.7	2.2 × 10^−4^	1.2 × 10^−2^
	*hsa-circCSNK1G3-001*	1.3 × 10^−6^	−1.1	0.29	14.1	1.8 × 10^−4^	1.2 × 10^−2^
	*hsa-circRNF138-001*	4.8 × 10^−7^	2.4	0.87	13	3.1 × 10^−4^	1.4 × 10^−2^
	*hsa-circLINC00632-001*	1.4 × 10^−6^	−1	0.28	12.7	3.7 × 10^−4^	1.4 × 10^−2^
	*hsa-circEMILIN2-001*	1.2 × 10^−6^	−1	0.31	11.3	7.9 × 10^−4^	2.7 × 10^−2^
Decidual tissue	*hsa-circADAMTS6-006*	2.2 × 10^−6^	−2.2	0.22	130.7	2.9 × 10^−30^	2.4 × 10^−27^
	*hsa-circEMILIN2-001*	2.2 × 10^−6^	−1.7	0.2	85.6	2.2 × 10^−20^	9.4 × 10^−18^
	*hsa-circRHOBTB3-006*	1.9 × 10^−6^	−1.8	0.21	79.8	4.1 × 10^−19^	1.2 × 10^−16^
	*hsa-circFCHO2-003*	1.9 × 10^−6^	−1.7	0.21	78.1	9.9 × 10^−19^	2.1 × 10^−16^
	*hsa-circCDYL-001*	4.6 × 10^−6^	−1	0.12	66.2	4.1 × 10^−16^	6.8 × 10^−14^
	*hsa-circMORC3-001*	3.6 × 10^−6^	−1	0.14	54.9	1.2 × 10^−13^	1.8 × 10^−11^
	*hsa-circZNF124-002*	1.9 × 10^−6^	−1.2	0.2	40.5	1.9 × 10^−10^	2.3 × 10^−8^
	*hsa-circSLC8A1-002*	4.6 × 10^−6^	−0.7	0.12	39.3	3.7 × 10^−10^	3.9 × 10^−8^
	*hsa-circCDYL2-001*	1.6 × 10^−6^	−1.2	0.22	34.8	3.6 × 10^−9^	3.1 × 10^−7^
	*hsa-circSEC62-001*	7.9 × 10^−7^	−1.8	0.34	33.8	6.1 × 10^−9^	4.7 × 10^−7^
	*hsa-circBTBD10-002*	1.8 × 10^−7^	−5.5	1.21	33	9.2 × 10^−9^	6.4 × 10^−7^
	*hsa-circGREB1L-001*	4.8 × 10^−7^	2.4	0.5	31.7	1.8 × 10^−8^	1.1 × 10^−6^
	*hsa-circFAM114A2-004*	1.2 × 10^−6^	−1.3	0.26	30.4	3.5 × 10^−8^	2.1 × 10^−6^
	*hsa-circSLF2-001*	7.6 × 10^−7^	−1.7	0.34	29.4	5.9 × 10^−8^	3.1 × 10^−6^
	*hsa-circTTC39C-001*	1.1 × 10^−6^	−1.4	0.26	29.4	5.8 × 10^−8^	3.1 × 10^−6^
	*hsa-circHOOK3-001*	4.7 × 10^−7^	−2.2	0.48	28.4	1.0 × 10^−7^	5.0 × 10^−6^
	*hsa-circELK4-001*	1.6 × 10^−6^	−1.1	0.21	27.4	1.6 × 10^−7^	7.7 × 10^−6^
	*hsa-circARHGAP10-001*	9.0 × 10^−7^	−1.4	0.3	25.6	4.3 × 10^−7^	1.9 × 10^−5^
	*hsa-circESYT2-001*	1.9 × 10^−6^	−1	0.19	25.5	4.5 × 10^−7^	1.9 × 10^−5^
	*hsa-circTUBGCP3-001*	1.8 × 10^−7^	−4.4	1.22	24.4	7.7 × 10^−7^	3.1 × 10^−5^

## Data Availability

Expression data and descriptive data of the detected circRNAs are available in Supplementary Materials S1 and S2, respectively.

## Supplementary Materials

The supporting information can be downloaded at: https://www.mdpi.com/article/10.3390/ijms252312622/s1.
