# Supplementary material for: The circRNA Landscape in Recurrent Pregnacy Loss (RPL): A Comparison of Four Reproductive Tissues
Source: Int J Mol Sci. 2024 Nov 25;25(23):12622. doi: 10.3390/ijms252312622 (PMC11641099; doi:10.3390/ijms252312622)
Supplement: Supplementary file 1 [file ijms-25-12622-s001.zip › Captions.pdf]

Supplementary figure 1. Plots depicting some characteristic circRNA properties. A) Position of the circRNAs identified in each chromosome B) Number of exons inside each circRNA

Supplementary figure 2. Bar plot with the result of the conservation analysis. In the x-axis the different categories described in "Material and methods" and in the y-axis the number of circRNAs in each category.

Supplementary figure 3. Biological processes described in circRNA-based functional analysis detected in decidual immune cells. If the same gene appears in different functions these are connected with blue lines. The size of the node depends on the number of genes summed by each term (the more genes the more size) and the color of the node indicates the degree of FDR significance (from 0 to 0.05).

Supplementary figure 4. Biological processes described in circRNA-based functional analysis detected in villus tissue. If the same gene appears in different functions these are connected with blue lines. The size of the node depends on the number of genes summed by each term (the more genes the more size) and the color of the node indicates the degree of FDR significance (from 0 to 0.05).

Supplementary table 1. Sample information and summary of the alignment to the human reference genome (GRCh38).

Supplementary table 2. List of all differentially expressed circRNAs in the four tissues and their baseMean, log2FoldChange, lfsSE, stat, p-value and p-adjusted values.

Supplementary material 1. Expression matrix from CIRIquant for the selected circRNAs.

Supplementary material 2. List of detected circRNAs with DCC and CIRI2 and their position, origin gene information and conservation status and CIRCpedia ID.
