# Supplementary material for: The circRNA Landscape in Recurrent Pregnacy Loss (RPL): A Comparison of Four Reproductive Tissues
Source: Int J Mol Sci. 2024 Nov 25;25(23):12622. doi: 10.3390/ijms252312622 (PMC11641099; doi:10.3390/ijms252312622)
Supplement: Supplementary file 1 [file ijms-25-12622-s001.zip › Supplementary Table S1.pdf]

Table S1: Sample information and summary of the alignment to the human reference genome (GRCh38).

Project ID	Total Reads (millions) Mean ± SD	Average reads after filtering (%)*1	Average rRNA reads (%)*2	Average of uniquely aligned reads (%)*3	Average of multi-aligned reads (%)*3	Average of chimeric reads (%)*3
PRJNA760763	53,12 ± 7,45	94,51%	0,75%	94,63%	4,50%	0,39%
PRJNA813430	41,74 ± 1,81	97,63%	1,81%	94,15%	4,43%	0,59%
PRJNA314429	31,57 ± 1,61	94,90%	7,56%	93,41%	5,93%	0,26%
PRJNA819201	48,30 ± 1,96	94,19%	1,58%	95,22%	4,05%	0,26%

*1 Percentage of reads surviving trimming by Trimmomatic when compared to raw reads. Calculated from average values from each tissue.

*2 Percentage of rRNA reads when compared to those that survided trimming. Calculated from average values from each tissue.

*3 Percentages calculated when compared to reads surviving trimming and rRNA removal. Calculated from average values from each tissue.
