# Supplementary figures and images for: The circRNA Landscape in Recurrent Pregnacy Loss (RPL): A Comparison of Four Reproductive Tissues
Source: Int J Mol Sci. 2024 Nov 25;25(23):12622. doi: 10.3390/ijms252312622 (PMC11641099; doi:10.3390/ijms252312622)

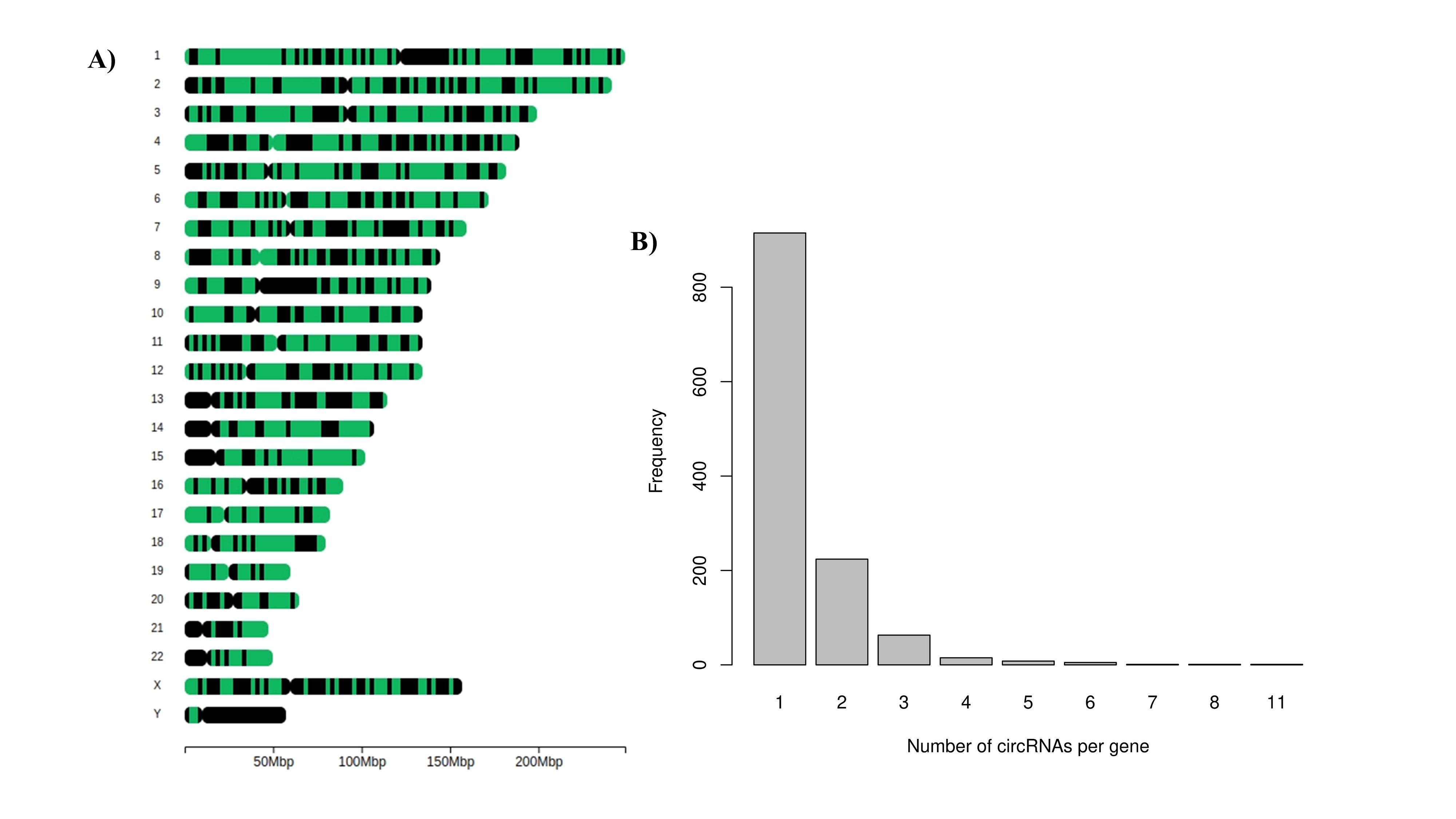

Supplement: Supplementary file 1 [file ijms-25-12622-s001.zip › Supplementary Figure S1.jpg]

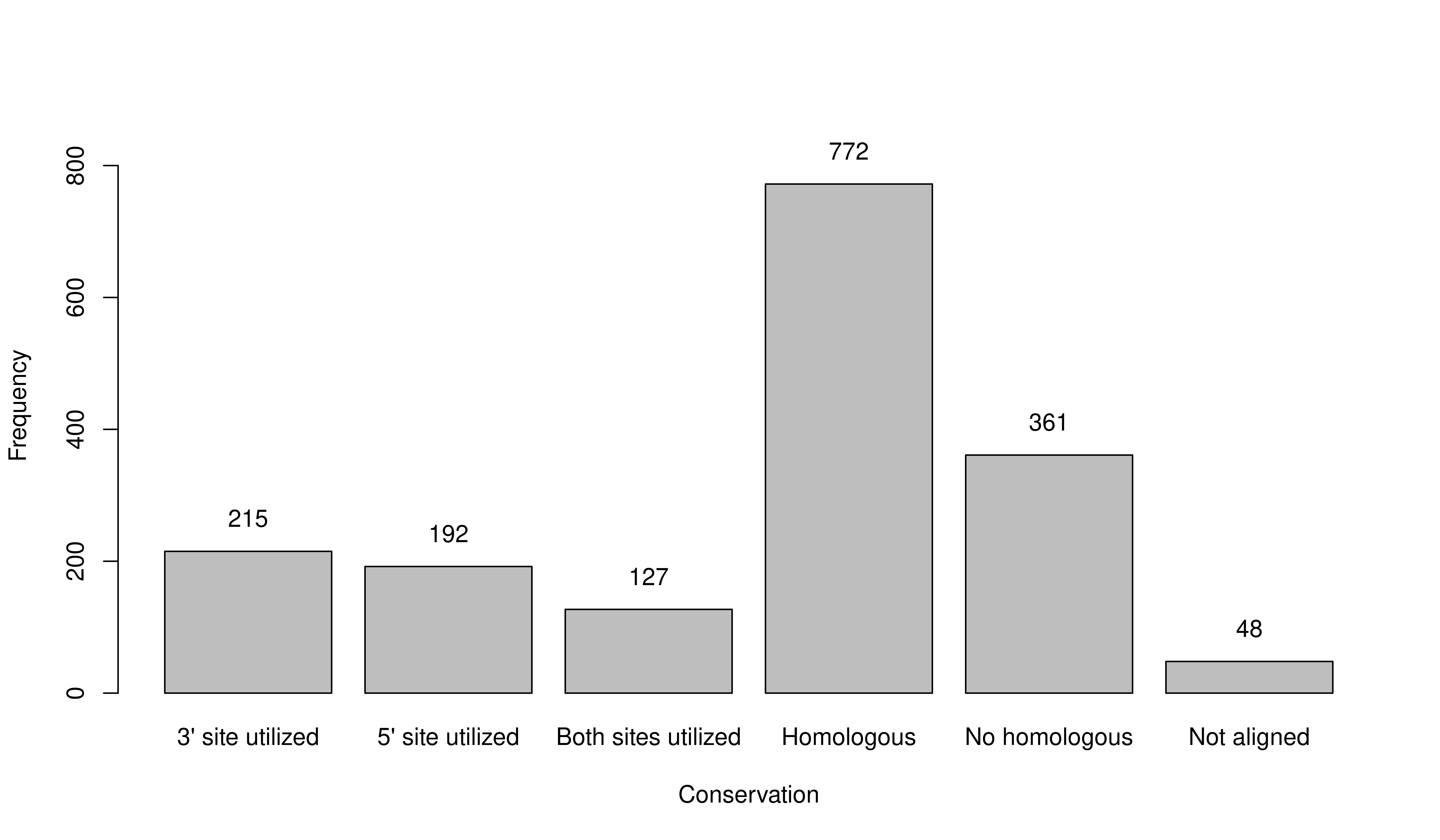

Supplement: Supplementary file 1 [file ijms-25-12622-s001.zip › Supplementary Figure S2.tiff]

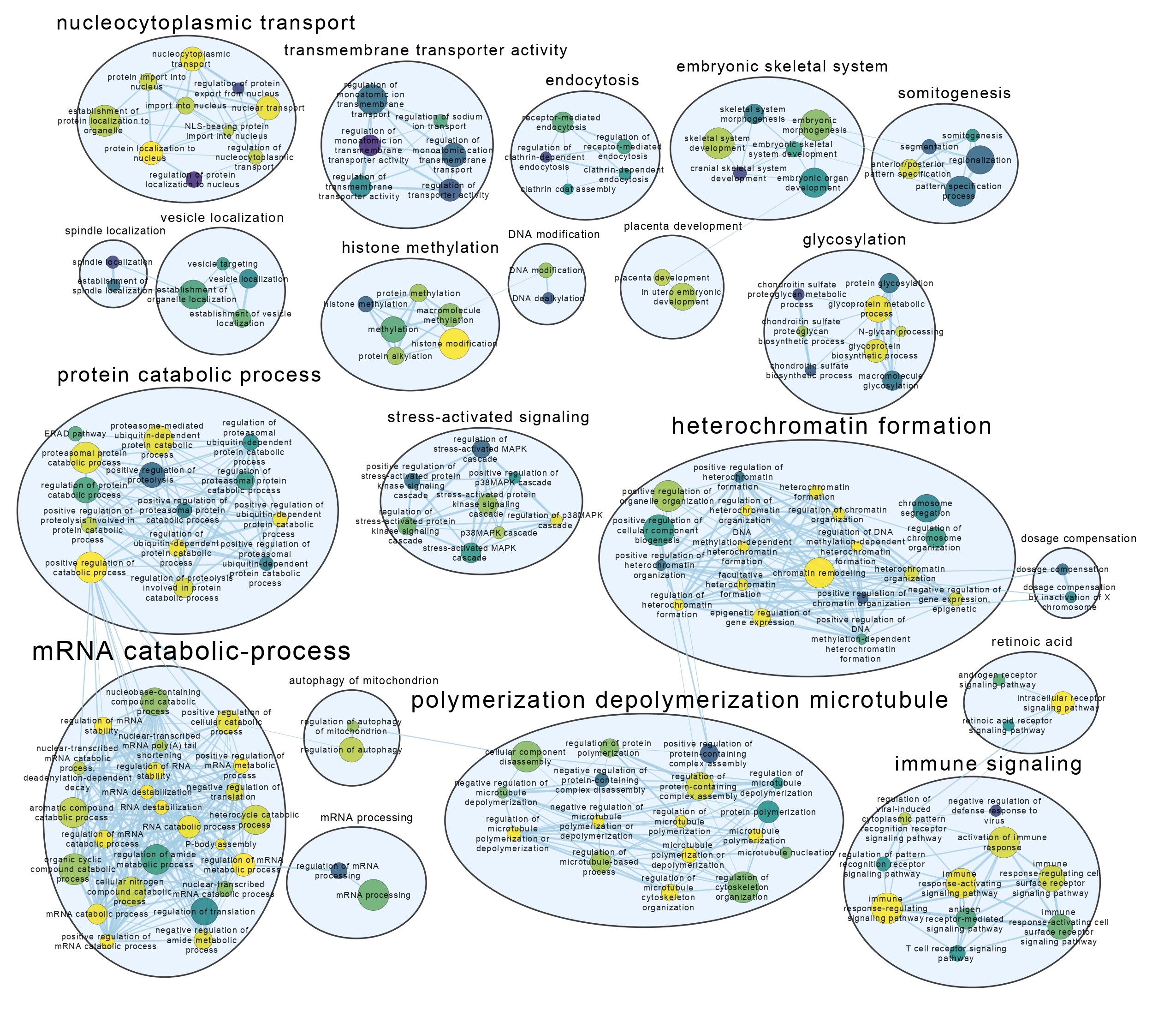

Supplement: Supplementary file 1 [file ijms-25-12622-s001.zip › Supplementary Figure S3.jpg]

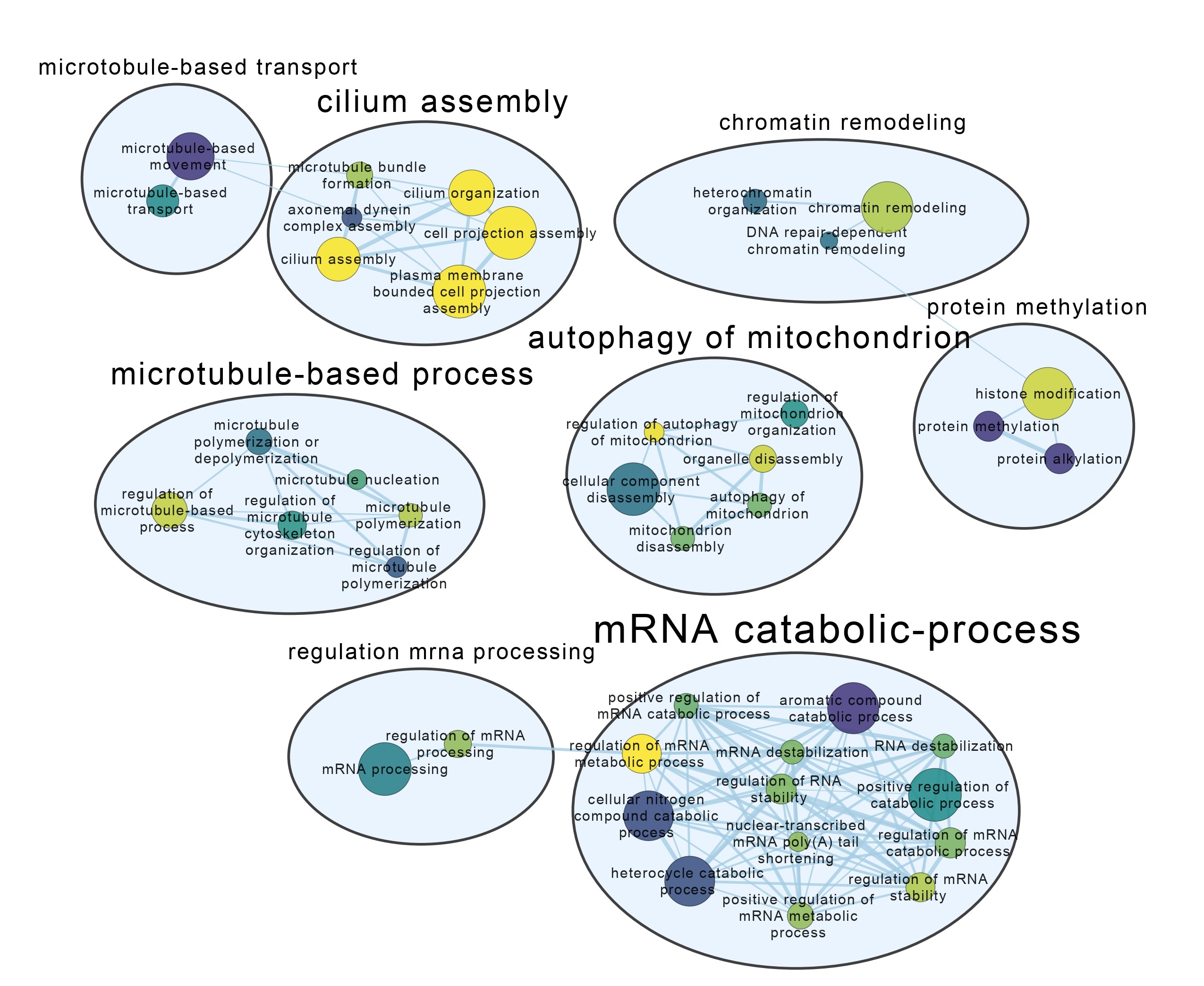

Supplement: Supplementary file 1 [file ijms-25-12622-s001.zip › Supplementary Figure S4.jpg]
